# Three *de novo* assembled wild cacao genomes from the Upper Amazon

**DOI:** 10.1038/s41597-024-03215-1

**Published:** 2024-04-11

**Authors:** Orestis Nousias, Jinfang Zheng, Tang Li, Lyndel W. Meinhardt, Bryan Bailey, Osman Gutierrez, Indrani K. Baruah, Stephen P. Cohen, Dapeng Zhang, Yanbin Yin

**Affiliations:** 1 Nebraska Food for Health Center, Department of Food Science and Technology, University of Nebraska-Lincoln, Lincoln, NE USA; 2 U.S. Department of Agriculture, Sustainable Perennial Crops Laboratory, Beltsville, MD USA; 3 U.S. Department of Agriculture, Subtropical Horticulture Research Station, Miami, FL USA

**Keywords:** Genetic variation, Plant evolution

## Abstract

*Theobroma cacao*, the chocolate tree, is indigenous to the Amazon basin, the greatest biodiversity hotspot on earth. Recent advancement in plant genomics highlights the importance of *de novo* sequencing of multiple reference genomes to capture the genome diversity present in different cacao populations. In this study, three high-quality chromosome-level genomes of wild cacao were constructed, *de novo* assembled with HiFi long reads sequencing, and scaffolded using a reference-free strategy. These genomes represent the three most important genetic clusters of cacao trees from the Upper Amazon region. The three wild cacao genomes were compared with two reference genomes of domesticated cacao. The five cacao genetic clusters were inferred to have diverged in the early and middle Pleistocene period, approximately 1.83–0.69 million years ago. The results shown here serve as an example of understanding how the Amazonian biodiversity was developed. The three wild cacao genomes provide valuable resources for studying genetic diversity and advancing genetic improvement of this species.

## Background & Summary

Cacao (*Theobroma cacao* L.) is an evergreen tree native to the Amazon rainforests^1,2^. Cacao is a diploid (2n = 2x = 20) species in the *Malvaceae* family. Among the 22 known species in the genus *Theobroma*, *T. cacao* is the only one grown commercially for the production of seeds (beans), which serve as the raw material for chocolate production. Cacao farming predominantly takes place on small-scale farms located in tropical developing nations. It is estimated that five to six million small holder farmers are engaged in cacao farming worldwide, supporting the livelihoods of approximately 40 to 50 million people^3^. With a global production of 4.75 million metric tons of dry cacao beans (The International Cocoa Organization, 2020), the global chocolate industry had a retail market value of USD 106.2 billion in 2017 and is expected to grow to USD 189.9 billion by 2026^4^.

The domestication of cacao is believed to have occurred around 5,300 years ago^5^. It is proposed that domestication took place at multiple sites by different indigenous groups in tropical America^5–8^. The fermentation of sweet cacao for the production of an alcoholic beverage preceded the grinding of the seeds and may have played a crucial role in the initial development of cacao farming^5,7,8^. While cacao was initially classified into three genetic groups (Criollo, Forastero, and Trinitario), subsequent research suggests that cacao germplasms can be divided into ten distinct genetic clusters, including Amelonado, Contamana, Criollo, Curaray, Guianna, Iquitos, Marañon, Nacional, Nanay, and Purús^9^. Recent collecting expeditions in the Amazon have identified additional germplasm groups, expanding our understanding of cacao’s genetic diversity^10,11^. These natural populations represent the primary gene pool of cacao in the Amazon basin, spanning from French Guiana to Bolivia^2,12,13^. Consequently, cacao is among the tropical plants with natural populations distributed throughout the entire Amazon region. These populations exhibit differentiation and adaptation to diverse environmental conditions, making cacao an ideal species for studying the spatial distribution of genetic diversity in tree species within the Amazon.

The hypothesis of Pleistocene refugia has been proposed to explain the significant intra-specific divergence of T. cacao in the Amazon^13,14^. According to this theory, the glacial cycles of the Pleistocene period caused forest cover to contract to limited areas or refuges, leading to the isolation of populations within climatically suitable regions and facilitating allopatric differentiation^15^. However, research by Motamayor *et al*. (2008) has shown that the geographical distribution of cacao genetic clusters does not correspond to the proposed refuge centers for other species in the region^16,17^. Instead, the differentiation patterns observed in the studied populations appear to align with potential dispersal barriers created by ancient ridges or palaeoarches^9^. Apart from the conflicting hypotheses explaining the geographical distribution of cacao populations, the timing of intra-specific divergence has also been a subject of debate. Richardson *et al*.^18^ analyzed dated phylogenies of chloroplast and nuclear DNA sequences and demonstrated that cacao diverged from its most recent common ancestor approximately 9.9 million years ago during the mid-to-late Miocene^18^. This suggests that cacao had ample time to generate genetic diversity within the species. In the same study, two individuals of *T. cacao* formed a monophyletic group with stem and crown node ages estimated at 6.5 million years ago and 1.2 million years ago, respectively^18^. Conversely, Thomas *et al*.^14^ proposed that the last glaciation period (22,000 to 13,000 years BP) had the most significant pre-human impact on the present distribution and diversity of cacao^14^.

The genome size of cacao is estimated to be between 430 and 445 Mbp based on reported reference genome assemblies of the two domesticated populations, namely ‘B97-61/B2’ from the Criollo group and ‘Matina 1–6’ from the Amelonado^19–21^. These reference genomes have provided valuable resources for recent studies on the origin, evolution, and adaptation of cacao^22–24^. They have also facilitated the development of genomic tools for breeding new cacao varieties with improved yield, tolerance to biotic and abiotic stress, and desired quality attributes^25–29^.

However, in recent years, increased plant genome projects have revealed significant genomic variation among related individuals. This high degree of genomic variation highlights the importance of *de novo* sequencing of multiple reference genomes to capture the genome diversity present within a given plant species^30,31^. Furthermore, the two cacao varieties with published reference genomes (Criollo and Amelonado) are self-compatible, and represent domesticated varieties from Mesoamerica and East Amazonia, respectively, and are far removed from the putative origin and center of genetic diversity for cacao. Recently, thirty-one high-quality genome assemblies were reported from four genetic populations (Iquitos, Nanay, Marañon, and Guiana)^23^. These genomes were *de novo* assembled into contigs using short reads and then scaffolded using a reference-based approach (with the Matina 1–6 reference genome), resulting in assembly sizes smaller (ranging from 30 to 70 Mb) than the estimated k-mer sizes, suggesting sequence data was lost due to the lack of reference-free scaffolding.

**Fig. 1:**
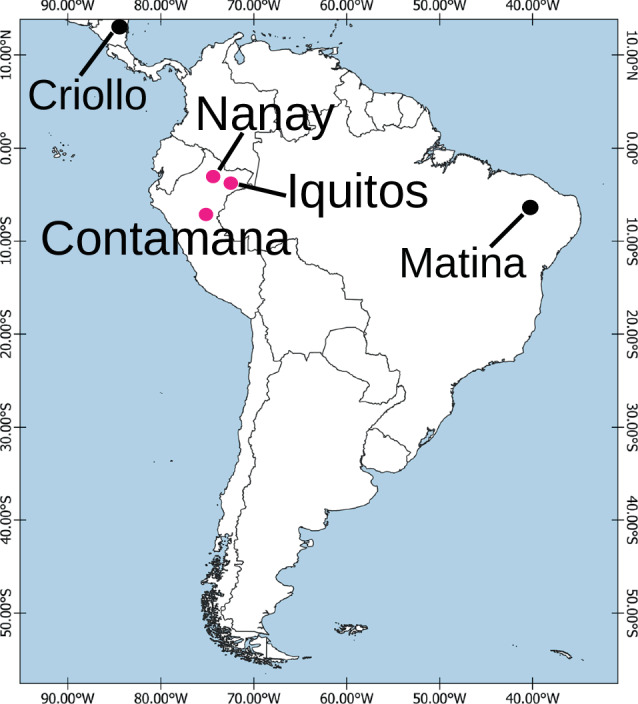
Geographic origins of the five cacao populations. The source populations of the three wild accessions sequenced in this paper (Nanay, Iquitos, and Contamana) are labeled in pink color.

In this study, we present three high-quality *de novo* genome assemblies and their annotations for wild cacao populations originating from the Upper Amazon region (Contamana, Iquitos, Nanay, Fig. 1). We produced completely *de novo* chromosome-scale genome assemblies using long reads and Hi-C technology for comparative genomics research in cacao. By analyzing the genomic distance based on single-copy orthologous genes, we provide new insights into the timing of the intra-specific divergence of cacao. This study provides valuable genomic resources for cacao, serving as essential references for future research on the conservation and utilization of cacao genetic diversity.

## Materials and Methods

### Plant material and long read sequencing

Leaf samples of *T. cacao* clones “SCA 6” (Contamana population), “IMC 67” (Iquitos population), and “POUND 7” (Nanay population) were collected from the greenhouse of USDA-ARS Sustainable Perennial Crops Laboratory (SPCL), Beltsville, Maryland. These three wild cacao clones were originally collected from the Peruvian Amazon (indicated in the map in Fig. 1), the putative center of origin of *T. cacao*. DNA samples were sequenced on PacBio Sequel II 8 M SMRT cells generating 42.6, 38.1, 40.7 gigabases of data for Contamana, Iquitos, and Nanay, respectively, corresponding to more than 100X coverage of each genome. The PacBio HiFi reads were used as an input to Hifiasm1 v0.15.4-r347^32,33^ with default parameters. BLAST results of the Hifiasm output assembly against the nt database were used as input for blobtools2 v1.1.1^34^ and scaffolds identified as possible contamination were removed from the assembly. Finally, purge_dups v1.2.5^35^ was used to remove haplotigs and contig overlaps.

### Chromatin conformation capture and sequencing

For each of the three cacao varieties, the Omni-C technique from Dovetail Genomics was employed to perform chromatin conformation capture^36^. In the process, chromatin was initially fixed using formaldehyde within the cell nucleus, followed by extraction. The fixed chromatin underwent DNAse I digestion, after which the ends of the chromatin were repaired and connected to a biotinylated bridge adapter. This was succeeded by a proximity ligation of ends containing the adapter. Post-proximity ligation, the crosslinks were undone, and the DNA was subsequently purified. The purified DNA was then subjected to a biotin removal process, specifically targeting biotin not incorporated within the ligated fragments. Sequencing libraries were created using NEBNext Ultra enzymes from New England Biolabs and adapters compatible with Illumina. Fragments containing biotin were separated using streptavidin beads, followed by PCR amplification of each library. The sequencing of the library was conducted on an Illumina HiSeqX system. Finally, only reads with a mapping quality (MQ) greater than 50 were selected for use in scaffolding.

### *de novo* scaffolding the assembly with HiRise

The *de novo* genome assembly from HiFi long reads and Dovetail OmniC library reads were used as input data for HiRise v2.0.5, a software pipeline designed specifically for using proximity ligation data to scaffold genome assemblies^37^. Dovetail OmniC library sequences were aligned to the draft input assembly using bwa v0.7.11^38^. The separations of Dovetail OmniC read pairs mapped within draft scaffolds were analyzed by HiRise to produce a likelihood model for genomic distance between read pairs, and the model was used to identify and break putative misjoins, to score prospective joins, and make joins above a threshold.

### Repeat region annotation and comparisons

Repeat regions were identified and annotated with a combination of homology and *ab initio* methods using RepeatModeler v2.0.2^39^, RepeatMasker v4.1.3^40^, and EDTA v2.0.0^41^ on the five cacao genomes. With RepeatMasker, repeats are assigned to different transposable element (TE) families and classes.

**Fig. 2:**
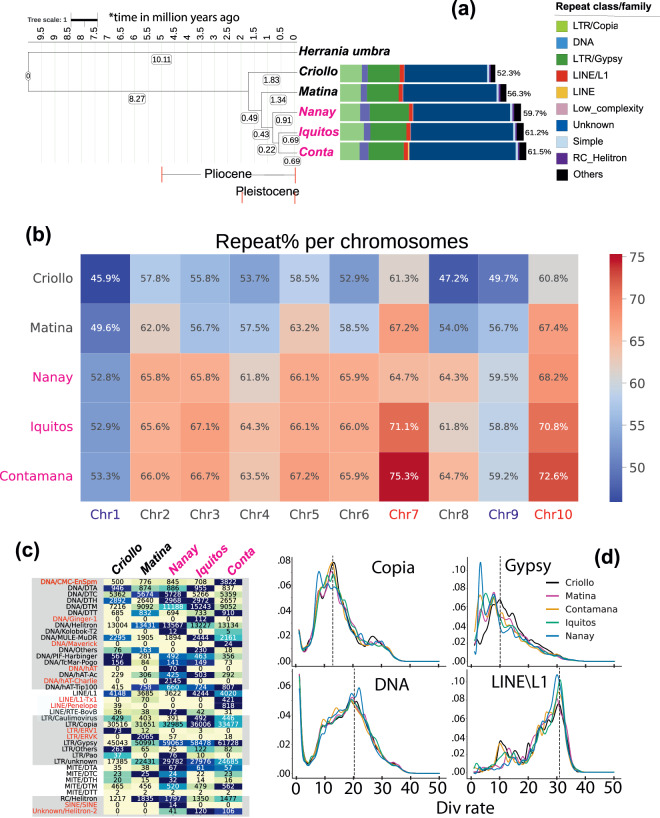
Repeat content comparisons among the five cacao genomes. (**a**) On the left is the species tree inferred by a super-alignment of 6,630 single-copy orthologous protein sequences from OrthoFinder. Bootstrap support is at 100 for all nodes. In addition to the five cacao genomes, Herrania umbratica is included as an outgroup. MCMCtree was used to infer the species divergence time. On the right is the bar plot of repeat content comparison of major transposable element (TE) classes. (**b**) Repeat content percentages in the 10 chromosomes of the five genomes. Repeats in all genomes were inferred using the same methods. (**c**) Comparison of repeat lengths (in kilobase pairs) at the TE family level among the five genomes. TE families are sorted according to their classes. (**d**) Kernel density (y axis) of sequence divergence rate (x axis) comparisons of the four most abundant TE families (all DNA families together as the DNA class). The Div rate is calculated as Kimura distance between each cacao repeat and their best Arabidopsis repeat match in the repeat library of RepeatMasker. Lower Div rate means the repeats are more similar between cacao and Arabidopsis or have diverged less after the two species separated.

The repeat content rates were compared among the five cacao genomes and stratified by chromosomes and TE classes/families (Fig. 2). Overall, the genomes of the three wild cacao varieties have higher repeat contents (61.5%, 61.2%, and 59.7% of the total genome lengths for Contamana, Iquitos, and Nanay, respectively, Fig. 2a) than the genomes of the two domesticated varieties (52.3% and 56.3% for Criollo and Matina, respectively). This may be because the domestication through artificial selection has purged the repetitive elements in the two genomes. These large differences of repeat contents among the five genomes also suggest that they have diverged early in evolution (species tree in Fig. 2a,see details later).

The repeat content profile also varies significantly among chromosomes of the three new genomes and the two reference genomes (Fig. 2b). In all the five genomes, Chr7 and Chr10 tend to have higher repeat contents than other chromosomes; the two chromosomes also vary the most among the five genomes along with Chr6 and Chr8 (>12% difference between the lowest Criollo and the highest Contamana). Chr1 and Chr9 have the lowest repeat contents in the five genomes. These inter-chromosomal variations in terms of repeat contents suggest the evolutionary selection pressure varies on different chromosomes influencing TE repeat content.

Breaking down the repeat contents into TE classes revealed that the three new genomes have higher percentages of long terminal retroelement (LTR) Gypsy repeats (dark green bars in Fig. 2a) as well unclassified TE repeats (dark blue bars) than Criollo and Matina. Further looking at the TE families (Fig. 2c) under each class identified some genome-specific TE families, such as hAT, hAT-Charlie and SINE in Nanay, Ginger-1 in Iquitos, and Maverick in Contamana. There are also TE families significantly expanded in some genomes, such as CMC-EnSpm, L1-Tx1 and Penelope in Contamana, ERV1 and ERVK in Criollo and Matina, Helitron-2 in Iquitos and Contamana.

To study the divergence rate of different TEs, we used the RepeatMasker utility scripts to calculate the kimura distances between the repeats of each TE family in Cacao genomes and the *Arabidopsis thaliana* reference repeat sequences. The *Arabidopsis* repeat sequences are provided by RepeatMasker and chosen at the command line aligning cacao repeats with them. Next we used parseRM.pl^42^ (https://github.com/4ureliek/Parsing-RepeatMasker-Outputs) to parse the repeat sequence alignments and get the bins of the Kimura distances. These bins contain the counts of the repeat elements of each TE family with the Kimura distance in the range of the bin (e.g., [0,0.1]). We compared the divergence rates of different TE families (between cacao and *A. thaliana*) by plotting their Kimura distance distributions. All analyses were performed with custom python scripts.

Kimura distance for each repeat pair indicates the divergence rate after Criollo and Arabidopsis separated from each other. Taking all the repeat pairs of each TE class and their Kimura distances to make plots (Fig. 2d), we found that the LTR (Copia + Gypsy) repeat distributions had a peak at a lower divergence rate than the peak of DNA and LINE\L1 repeat distributions. This suggests that LTR repeats have evolved slower than other TE elements since the divergence of cacao and Arabidopsis. Comparing the five cacao genomes also showed that the most abundant repeat family gypsy in Criollo had evolved much faster that in the other cacao genomes (black curve in Fig. 2d with a kimura peak of a higher divergence rate). Compared to the other cacao genomes, Nanay had a peak of lower divergence rate in both Copia and Gypsy plots (blue curve in Fig. 2d). Overall, this means the LTR repeats in the domesticated cacao especially Criollo evolved faster.

### Gene model prediction

To predict protein coding gene models from the three new cacao genomes, MAKER v3.01.04^43^ was utilized to predict protein-coding genes through a combination of *ab initio* and homology-based techniques. The software allows the use of transcriptome and protein evidence for homology-based gene discovery. In this study, *T. cacao* RNA-seq raw reads were retrieved from GenBank (PRJNA785999, PRJNA471714) and trimmed using Trim_galore v0.6.8^44^. The clean reads were then assembled into transcripts utilizing Trinity v2.15.0^45^. Furthermore, all protein sequences of *T. cacao* were downloaded from Phytozome^46^ and UniProt^47^, using cacao as a search term in the two databases. To facilitate *ab initio* gene prediction, MAKER merged SNAP v0.0.0^48^ and Augustus v3.4.0^49^ outcomes, and the process was iterated three times to enhance accuracy. During the first round, Trinity transcripts, *A. thaliana* protein sequences, and UniProt protein sequences were fed into MAKER. The resulting output was then used to train Augustus models with BUSCOs v4^50^ assistance and SNAP models using gene sequences from maker2gff. During the second round, MAKER employed the Augustus and SNAP models to predict *ab initio* genes, followed by another round of model training and running using the output from the second round. The third MAKER runs outcome was used as the input to PASA v2.5.2 and Evidence modeler v2.0.0^51,52^. The final gene models were annotated for protein functions with eggNOG-mapper v2.1.6^53^.

For the two reference genomes, we downloaded their gene models from GenBank: Criollo (GCF_000208745.1) and Matina 1–6 (GCF_000403535.1). In addition, we have also run MAKER on the two reference genomes using the same pipeline described above, in order to verify the disease resistance gene count difference observed in the five genomes (see below).

The BUSCO scores with the gene model input indicated that the gene model predictions were of high quality, ranging from 89.2% to 95.1% for our three cacao genomes (Table 1). Not surprisingly, they are lower than the two reference genomes, whose gene models have undergone continuous improvement in the last decade^19–21^. However, the Matina genome had a substantially higher number of predicted gene models (27,329) than the other four genomes. Moreover, the Matina genome displayed structural differences in the number of single exon genes, the number of overlapping genes, and the mean gene length compared to the other four genomes (Table 1).

**Table 1:** Gene model prediction statistics.

Gene model statistics	Contamana	Iquitos	Nanay	Criollo	Matina
Number of protein-coding genes	20,623	21,001	20,942	21,437	27,379
Number of genes overlapping	774	778	784	452	1,829
Number of single exon genes	2,073	3,851	3,817	3,423	5,726
Mean gene length (bp)	3,647	3,560	3,557	3,960	3,530
Gene prediction Busco scores	89.2%	95.1%	93.8%	99.5%	99.3%

### Intra-species divergence time estimation

We combined protein sequences of the five cacao genomes and the *Herrania umbratica* (GCF_002168275.1, as an outgroup) to define orthologous groups (orthogroups). Genome annotation files were processed to only keep the longest isoform protein of each gene. Proteins of the six genomes were combined as input to OrthoFinder v2.5.4^54^, which generates orthogroups with the alignment tool MMseqs. 2 v12-113e3^55^.

A total 6,630 orthogroups were identified by OrthoFinder. All of the single-copy orthogroups (containing a single copy of gene from each of the six genomes) were aligned with MUSCLE v5^56^. The alignments of the 6,630 single-copy orthogroups were concatenated into one super alignment. A phylogenetic tree was built using RAxMLv8^57^ to represent the species tree with 100 bootstraps and the evolutionary model -m PROTGAMMAJTT. The species tree (Fig. 2a) with *H. umbratica* as the outgroup shows that the Mesoamerican Criollo separated from the other cultivars the earliest, followed by the East Amazonian Amelonado (Matina 1–6), then the three wild cultivars. The alignment of single copy orthologs (amino acids) that was used to infer the species tree was converted to codon alignment using the nucleotide coding sequences of the single copy orthologs. With the concatenated codon alignments (one sequence for each of the six genomes) inferred by 6,630 single copy orthogroups, we estimated the divergence time for the five cacao genomes plus *H. umbratica* (the closest to Cacao among the 12 species) by MCMCtree^58^ with one calibration: the divergence time of *H. umbratica* and *T. cacao* = 9~12 MYA. The calibration time was obtained from the TimeTree database^59^. We performed 10 MCMCtree runs to ensure the confidence of the results. In all 10 runs, we used a high sampling rate of 10,000 and excluded the missing columns using the option from the.ctl file of MCMCtree set to “1”. From all 10 runs, the results were almost identical with the results shown in Fig. 2a.

The cacao speciation time from *Herrania umbratica* at 10 MYA inferred here (Fig. 2a) is in line with the literature^18,20,22^. Our results showed that the population divergence of the five cacao genetic clusters occurred during the Pleistocene epoch (within 2 MYA). Criollo population emerged at ~1.83 MYA, Matina population at ~1.34 MY, Nanay population at ~0.91 MY and Contamana and Iquitos populations at ~0.69 MY (Fig. 2a). This finding fits the established theory of the Neogene and Pleistocene origin of many neotropical species. It rejects the hypothesis that cacao population differentiation was caused by the Last Glacial Maximum (LGM) induced refugia (Thomas *et al*.^14^), which happened 24,000 ~15,000 years ago. Accurate estimation of divergence time is essential for understanding the evolutionary history of cacao and provides a framework for making future predictions about the effects of environmental change and human activities on its populations.

## Data Records

The raw PacBio HiFi reads (FastQ format) are available in the NCBI SRA database under the project number PRJNA982528 (Nanay: SRR25256512^60^, Contamana: SRR25256510^61^, Iquitos: SRR25256511^62^), so are the raw Omni-C short reads in FastQ format (Nanay: SRR28464384^63^, Iquitos: SRR28464385^64^, Contamana: SRR28464201^65^). The genome assemblies (Fasta format of DNA and protein sequences) and annotations (GFF format) are available at FigShare^66^ and https://bcb.unl.edu/USDA_genomes_CACAO/.

In addition, the transposable element and repeat annotation (plain text format from RepeatMasker), the assemblies (Fasta format) of the three cacao genomes and gene model annotations (GFF format), the protein function annotation (TSV format from eggNOG-mapper), and the structural variation were deposited in FigShare^66^ with the DOI number 10.6084/m9.figshare.25066010.v1. The three new cacao genome assemblies can be also found in GenBank: Iquitos GCA_958328385.1^67^, Nanay GCA_958329735.1^68^, Contamana GCA_958329045.1^69^.

## Technical Validation

**Fig. 3:**
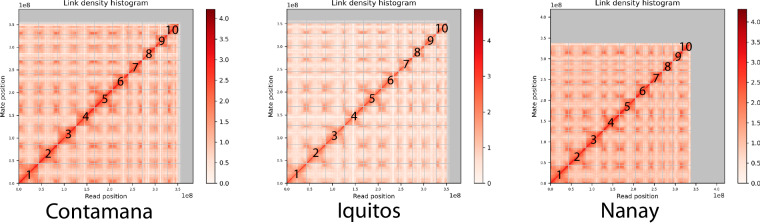
HiRise scaffolding linkage density histograms of the three cacao genome assemblies. The two axes are the positions (in base pairs) of paired Omni-C reads mapped in the genome assembly. The grids separate the major linkage groups corresponding to the 10 pseudo-chromosomes. The gray areas contain scaffolds that are not placed in the 10 pseudo-chromosomes.

The HiRise linkage density plots of the three new cacao genomes (Fig. 3) revealed 10 chromosomes, the same number as the two reference genomes (Criollo and Matina). The final assemblies had N50 values of 39.46, 39.49, and 34.43 Mbp for Contamana, Iquitos, and Nanay, respectively (Table 2). Compared to the Criollo and Matina reference genomes, Contamana and Iquitos genome assemblies showed better quality with higher N50, L50, N90, and L90 values (Table 2). The genome BUSCO^50^ scores (using eukaryote_odb10) were also slightly better in Nanay, Iquitos, and Contamana, than in the two reference genomes (Table 2).

**Table 2:** Assembly statistics of the three new cacao genomes and the two reference genomes.

Assembly	Contamana	Iquitos	Nanay	Criollo**	Matina 1–6**
Total HiFi long reads (Gigabases)	21.4	19.1	20.4	—	—
Number of scaffolds	175	170	1895	431	711
Total genome length	383,477,156	381,649,652	419,275,778	324,879,930	345,993,675
Length of the 10 chromosomes	352,058,514	351,354,296	338,407,810	314,189,522	330,456,197
GC (%)	33,79	33.8	34.9	32,1	32,5
N50	39,464,999	39,489,205	34,430,447	36,364,294	34,397,752
L50	5	5	6	5	5
N90	24,455,724	23,705,257	41,995	21,614,486	21,543,242
L90	10	10	580	9	10
k-mer completeness (%)	86.4	82.9	87.6	92.0	74.5
k-mer consensus QV (quality value)	61.0	61.6	53.0	38.9	32.4
BUSCO *	98.4%	98.8%	98.4%	98.1%	98.3%

*BUSCO scores are calculated with genome sequences as input using eukaryota_odb10.

^**^Criollo (GCF_000208745.1) and Matina 1–6 (GCF_000403535.1) genomes are downloaded from GenBank.

In addition, Merqury v1.4.1 was run on the DNA reads and genome assemblies of the five cacao cultivars to perform k-mer-based analyses^70^. K-mers shared by the sequencing reads and the genome assembly can be used to calculate the k-mer completeness (recovery rate). K-mers uniquely found in the genome assembly and absent in the sequencing reads can be considered as assembly consensus errors and used to calculate the assembly base-pair quality value (QV). To run Merqury, we used HiFi reads of Nanay, Iquitos, and Contamana that we sequenced (see SRA IDs above). We downloaded Illumina NovaSeq. 6000 reads (SRR21562109) for Criollo, and 454 GS FLX + reads (SRR866472, SRR866474, SRR866481, SRR866483, SRR866484, SRR866485, SRR866487, SRR866488) for Matina. These reads were originally used to build the Criollo and Matina reference genomes.

Comparing the five genomes, Criollo has the highest k-mer completeness at 92.0%, while Matina has the lowest at 74.5% (Table 2). The three new genomes have k-mer completeness (82.9~87.6%) better than Matina but lower than Criollo. For assembly QVs, all the three new genomes have much higher values (53.0~61.6) than Criollo and Matina. Contamana stands out with a QV at 61.0 and a completeness score at 86.4%. Criollo has a much lower QV at 38.8. Matina has QV at 32.4 being the lowest among the five genomes. Lastly, the HiFi reads were quality assessed to obtain the average Phred scores using FastQC v0.12^44^, which confirms that Nanay reads exhibit a lower Phred score (average score of 60.5) compared to Iquitos (69) and Contamana (71.1). However, in general the average quality of all HiFi reads is outstanding. This supports orthogonally the initial k-mer analysis findings.

These k-mer-based assessments underscore the high-quality nature of the three new cacao genome assemblies, which are generally better than the two reference genomes. Despite the inherent challenges in genome assembly, these QV scores and completeness percentages highlight their reliability in genomic data analysis.

Nanay, compared to the other genomes, has a lower genome assembly quality (many more scaffolds, much lower N50 and N90, Table 2). Its much larger total genome length is probably due to the much larger number of unplaced scaffolds in the chromosomes (Fig. 3), although its BUSCO score, QV, and k-mer completeness are comparable to the other genomes. If only consider the ten pseudo-chromosomes, the total genome length was 352.06 Mbp for Contamana, 351.84 Mbp for Iquitos, and 338.4 Mbp for Nanay. In contrast, the total chromosome length of Criollo and Matina was 314.18 Mbp and 330 Mbp, respectively.

In summary, the three new wild cacao genomes, like other recent cacao genome sequencing studies^23,71^, will further our understanding of cacao’s genetic diversity and evolution.

## Data Availability

No code was developed for implementing a software.
