# Author Correction: Three *de novo* assembled wild cacao genomes from the Upper Amazon

**DOI:** 10.1038/s41597-024-03545-0

**Published:** 2024-06-25

**Authors:** Orestis Nousias, Jinfang Zheng, Tang Li, Lyndel W. Meinhardt, Bryan Bailey, Osman Gutierrez, Indrani K. Baruah, Stephen P. Cohen, Dapeng Zhang, Yanbin Yin

**Affiliations:** 1 Nebraska Food for Health Center, Department of Food Science and Technology, University of Nebraska-Lincoln, Lincoln, NE USA; 2 U.S. Department of Agriculture, Sustainable Perennial Crops Laboratory, Beltsville, MD USA; 3 U.S. Department of Agriculture, Subtropical Horticulture Research Station, Miami, FL USA

**Keywords:** Genetic variation, Plant evolution, Genetic variation, Plant evolution

Correction to: *Scientific Data* 10.1038/s41597-024-03215-1, published online 11 April 2024

In Fig. 1 of this article the geolocations of the Nanay, Iquitos, and Contamana populations were incorrectly shown; the figure has now been corrected. The original article has been updated.

